# The isothermal Boltzmann–Gibbs entropy reduction affects survival of the fruit fly *Drosophila melanogaster*

**DOI:** 10.1038/s41598-023-41482-x

**Published:** 2023-08-29

**Authors:** Iwona Gruss, Jacek Twardowski, Małgorzata Samsel-Czekała, Jarosław Beznosiuk, Czesław Wandzel, Kamila Twardowska, Rafal J. Wiglusz

**Affiliations:** 1 Department of Plant Protection, Wroclaw University of Environmental and Life Sciences, Grunwaldzki Sq. 24a, 50363 Wroclaw, Poland; 2 Institute of Low Temperature and Structure Research, Polish Academy of Sciences, Okolna 2, 50422 Wroclaw, Poland; 3 PER Poland S.A, Ul. Zygmunta Starego 9, 44100 Gliwice, Poland; 4 PER Switzerland AG, Landstrasse 151, 9494 Schaan, Liechtenstein

**Keywords:** Biological techniques, Developmental biology, Psychology, Physics

## Abstract

To the best of our knowledge, this is the first experimental evidence of the effect of isothermal changes in entropy on a living organism. In greater detail, the effect of the reduction of the total Boltzmann–Gibbs entropy (S) of the aquatic environment on the survival rate and body mass of the fruit fly *Drosophila melanogaster* was investigated. The tests were carried out in standard thermodynamic states at room temperature of 296.15 K and ambient atmospheric pressure of 1 bar. Two variants of entropy reduction (ΔS) were tested for ΔS = 28.49 and 51.14 J K^−1^ mol^−1^ compared to the blind and control samples. The entropy level was experimentally changed, using the quantum system for isothermal entropy reduction. This system is based on quantum bound entanglement of phonons and the phenomenon of phonon resonance (interference of phonon modes) in condensed matter (Silicon dioxide (SiO_2_) and single crystals of Silicon (Si^0^), Aluminum (Al^0^) plates (“chips”), glass, and water). All studied organisms were of the same age (1 day). Mortality was observed daily until the natural death of the organisms. The investigations showed that changes in the Boltzmann–Gibbs entropy affected the survival and body mass of the fruit flies. On the one hand, the reduction in entropy under isothermal conditions in the aquatic environment for ΔS = 28.49 J K^−1^ mol^−1^ resulted in an extension of the lifespan and an increase in the body mass of female fruit flies. On the other hand, the almost twofold reduction in this entropy for ΔS = 51.14 J K^−1^ mol^−1^ shortened the lives of the males. Thus, the lifespan and body mass of flies turned out to be a specific reaction of metabolism related to changes in the entropy of the aquatic environment.

## Introduction

The Boltzmann–Gibbs entropy is a thermodynamic state function related to the degree of randomness and disorder of a system and/or a dissipation of the energy. For a living organism, it is such a part of the energy that cannot be converted into work in the body. Changes in energy are necessary for all vital functions of the body, and excessive increases in entropy during certain developmental stages could be a negative phenomenon^1^. A living organism is a very complex and highly self-organized system^2^. It is an open, nonequilibrium system that exchanges entropy with the environment (transfers excessive entropy to the environment) in the form of degraded waste products, and, consequently, internal low-entropy states can be created^3^. Entropy is critical for all biological functions of the body, and its value changes significantly throughout life. An excessive level of system entropy in each development phase means disorganization of life functions and a decrease in the biological activity of all living organisms; therefore, every living organism tends to reduce its level^4^. In this case, an explanation of the basic mechanisms governing metabolic processes that occur in living organisms is required. Most importantly, the study of energy flow or transfers in the following aspects is desired: quantum, thermodynamic, chemical, electrical, magnetic, mechanical, and structural (fractal information, for example, protein and DNA)^5^.

Basic metabolic processes within a living cell are carried out in two types of biochemical reaction, namely catabolic (usually exothermic) and anabolic reactions (usually endothermic)^6^. In endotherms, all these reactions take place in a liquid medium, at stabilised temperature, only due to quantum catalytic interactions of specific enzymes (e.g., enzymatic proteins or RNA). In turn, in ectotherms, these reactions occur in some well-defined temperature ranges, specific to a given species^7,8^. Living organisms in intracellular catabolic (exothermic) processes generate appropriate amounts of Gibbs-free enthalpy, which is the basic source of energy in anabolic (endothermic) processes. Thus, it ensures the maintenance of an organised structure and lowering the level of internal entropy, especially that of water, which constitutes about 80% of the mass of the whole cell^9^. Quantum phenomena related to changes in the liquid medium are the subject of numerous investigations^10–12^. Transport of physical quantities, like energy, enthalpy, or entropy, can be mediated by appropriate particles (e.g., electrons and protons) or quasiparticles such as phonons. Moreover, quantum changes in the entropy are possible due to phonon interference (phonon resonance, quantum beat)^13^. The energy states of the liquid medium, resulting from the vibrational dynamics of its hydrogen bond network, were found to determine the resonant effects of quantum beats and tunnelling in all biochemical reactions catalyzed by enzymes^14–16^.

Optimisation of the entropy level simultaneously restores the biochemical balance between catabolism and anabolism by, for example, accelerating the autophagocytosis processes by activating the protein kinase enzyme, or activation of the alcohol dehydrogenase enzyme, and consequently counteracts the aging processes of living organisms, protecting them against diseases and prolonging their life^17^. Calorimetric analyses showed that the growth of microorganisms depends on changes in entropy^18,19^. Further analyses indicated that both the consumption of ‘negative entropy’ (products have higher entropy than substrates) and the generation of heat can be utilised by microorganisms to get rid of the excess production of internal entropy production, coming from homeostasis and growth^18^. The decrease in entropy was due to changes in the vibrational dynamics of the hydrogen bond network related to water molecules, which determine the resonance effects of quantum beats and tunnelling. The use of water with isothermally reduced entropy (its phonon and configurational parts) allows the reduction in the internal entropy of living organisms without generating additional Gibbs free energy for catabolic reactions. This phenomenon directly translates into an increase in the amount and availability of Gibbs free energy for all anabolic reactions^20,21^.

*Drosophila melanogaster* is an ectothermic organism; therefore, its lifespan depends on ambient temperature^22^. The organs involved in its metabolism, are primarily the fat body, oenocytes, the digestive tract, and the Malpighian tubules. The fat body can regulate metabolism, similar to adipose tissue and the adipocytokines it secretes in humans^23,24^. In turn, fly oenocytes behave similarly to hepatocytes, influencing lipid metabolism. It appears that the fat body of flies can store glycogen and its synthesis and decomposition, as the liver of mammals^23^. An indicator of the metabolic rate of insects is the fat body, which is the central storehouse of nutrients and energy reserves^24^. The adult stage of *D. melanogaster* may increase or decrease its body mass under the influence of a different diet^25^, or rearing at different temperatures, and a reduced body mass is associated with an increased metabolic rate^22^. In many investigations, flies are proposed as model organisms in the study of metabolic diseases^24,26^, which are affected by the level of entropy^27^. The other *Drosophila* species (*Drosophila simulans*) have been proposed as novel model organisms to study mitochondrial DNA ageing^28^. Furthermore, it has been shown that the health and ageing of organisms depend on mitochondrial quantum efficiency, including tunneling and coherence^29^.

The experiment was designed to investigate the response of a living organism to entropy reduction knowing that entropy affects life at a fundamental level (cellar)^1^ and that organisms tend to reduce^17^. The effect of lowering the entropy (two selected levels) of the liquid environment (medium) on the vital parameters of model animal organisms, *D. melanogaster,* was studied, in detail. In this way, the reduced entropy can affect organisms through the liquid food they consumed. This unique method has been used to reduce the total Boltzmann–Gibbs entropy (S) of the aquatic environment. It is the only known and safe method to reduce under isothermal (at room temperature) conditions (but without an external magnetic field or pressure) the total entropy (S) of living organisms, in this case, fruit flies (*Drosophila melanogaster*). The aim of the work was to investigate the influence of internal entropy reduction (without changing any external conditions mentioned above) on the rate of metabolism, determining the survival rate and body weight of the tested organisms. This was the first experiment in which we investigated the reduction in isothermal entropy in the above way on living organisms.

## Materials and methods

### The system to reduce the Boltzmann–Gibbs thermodynamic entropy in the liquid medium (Phononic Entropy Reducer, PER®)

In the experiment, the quantum system used for an isothermal entropy reduction PER® is based on quantum bound entanglement of phonons [^30–32^, and references therein] and on the phenomenon of phonon resonance (interference of phonon modes) in condensed matter (both silicon oxide, i.e. quartz (SiO_2_) and single crystals of silicon (Si^0^), aluminium (Alº), glass, and water). The technology was first applied to reduce the entropy of the liquid medium and, consequently, to increase the efficiency of enzymatic processes (enolase enzyme) by about 30%^30^. The phonon signals in this experiment were transferred remotely over a distance of 160 km (from Gliwice to Wroclaw), by two sets of quantum-entangled PER® chips-inductors, each set transmitted phonon modes from one of two types of a generator which were single crystals of SiO_2_ and Si^0^. As a result, chip-inductor No. 1 had in its phonon spectrum the interference component of phonon modes of a single crystal of quartz and, in turn, chip-inductor No. 2, that of a single crystal of silicon (Table 1). These modes, per the resonant interaction of waves generated because of interference (natural Fourier transform), caused resonance with appropriate types of vibrations, first with those in the walls of glass flasks and, finally, with those in the water contained in them. As a result, the vibration phase was synchronised, and the phonon entropy of water was lowered, which in turn caused a simultaneous isothermal reduction of the configurational entropy of water molecules and the network of hydrogen bonds of the entire liquid medium, in the whole space of glass flasks, in which the liquid food of the tested flies was placed. Chip inductor No. 2, generating only (three) modes of acoustic phonons from the generator-single crystal of Si (its standard entropy: S_0_ = 18.82 J K^−1^ mol^−1^) guaranteed a resonant interaction on the water medium (standard entropy of H_2_O: S_0_ = 69.96 J K^−1^ mol^−1^) significantly reducing its configurational and phonon entropy. In this case, a substantial reduction in entropy was caused by a considerable difference in the standard entropies of water and silicon, equal to ∆S = 51.14 J K^−1^ mol^−1^. Chip inductor 1, (SiO_2_), generating both acoustic and optical phonon modes, reduced the configurational and phonon entropy to a lesser extent than the previous, due to almost twofold lower entropy difference between water and quartz, amounting to ∆S = 28.49 J K^−1^ mol^−1^.

**Table 1:** Description of experimental treatments.

Number	Name	Description
1	SiO_2_	Standard entropy of the single crystal of quartz: S_0_ = 41,47 J K^−1^ mol^−1^
2	Si^0^	Standard entropy of the single crystal of silicon: S_0_ = 18,82 J K^−1^ mol^−1^
3	Blind test	A lack of quantum transfer of phonon modes from any single crystals, thus only the Al^0^ phonon modes may interact but these are in insignificant resonance with vibrations of H_2_O
4	Control test	Without external interference

### Experimental details

The whole experiment was carried out in a complete randomised block design with 5 blocks (Figure S1 [see Supplementary data]). The treatments were previously described in Table 1. One replication was a 50 ml glass flask containing an individual male or female of the adult fruit fly *Drosophila melanogaster*. The replications of the certain treatment were grouped into 6 flasks with an equal proportion of males (3 flasks) and females (3 flasks), further named as groups. The flasks were randomly distributed between the groups. The groups were limited by polystyrene walls and a bottom with dimensions: length 36.4 cm, width. 26.4 cm, and height 14.3 cm, and spaced 40 cm apart from one another. Such a solution ensures the insulation of groups from the influence of one another. Within each block, groups representing 4 treatments were randomised.

The total number of flasks and, at the same time, the total number of flies used in the test was 120 (60 females and 60 males, 15 individuals of each sex per treatment). Appropriate chip-inductors were attached to the outer wall of the flasks previously filled with the media.

### Animals model

The model organisms were fruit flies *Drosophila melanogaster* (Meigen, 1830) with confirmed species characteristics. Before starting the investigation, the flies were reared for several generations at a temperature of 23 °C (296.15 K), moderate light intensity in the photoperiod 12 h/12 h (day/ night) on the agar medium. To obtain one-age organisms, eggs were isolated and after hatching the larvae were grown to the pupal stage. At this stage, the pupae were transferred to separate test tubes and incubated until the emergence of adult flies, which were used for the test. Adults were identified by sex using stereomicroscopes. Before this, the flies were immobilised with anesthesia on ice for 2 min. The fitness of each individual was checked after anesthesia on only the viable individuals that were individually transferred to the test flasks.

In the experiment, a corn medium with the following composition was used: 1000 ml of distilled water, 8 g of agar, 67 g of sugar, 135 g of corn porridge and 100 g of yeast. The weight content of the water in the medium is 77%. The flies were incubated at a constant temperature of 23 °C ± 0.3 °C (296.15 ± 0.3 K) and the indoor humidity was 60%. Temperature and humidity were constantly monitored (measurements every 10 min) using the Extech RHT 10 m.

Flies during the experiment were incubated individually in 50 ml conical glass flasks. Each flask contained 20 ml of medium. The flasks were closed with cellulose stoppers. The flasks and stoppers were previously sterilised by autoclaving. The medium was unchanged during the experiment. All flies were transferred to the flasks simultaneously.

### Incubation of the model organism during the experiment

Survival and behaviour of the adult flies were observed from the first day after leaving the pupa to natural death with the frequency of once a day (12:00 + /− 1 h). The observations continued throughout the lifetime of the insects in all flasks. Dead flies were weighed on an analytical balance (accuracy up to 0.0001 g). The water loss was weighed once a week. The water was refilled with a metal medical needle in a volume of 1 ml per week. Additionally, each treatment provides 1 flask without any organism to control the water loss and a flask with a chip-inductor for refilling water.

### Data analysis

All statistical analyses of the data were performed using SAS University Edition software. To examine the differences in the average lifespan and average body weight of the flies in groups determined by experimental combinations, the F test of the analysis of variance in the block system was used. In addition to the combination, the blocking effect was checked in the model. In the case of significant differences in the means, Tukey's HSD test was used to determine homogeneous groups. The assumption of normality of data distribution was tested with the Shapiro–Wilk test and the quantile–quantile plot, implemented in the UNIVARIATE tool. Survival analysis was performed using the PROC LIFETEST tool. In its scope, the following were compared:

- Survival curves using the log-rank test and the Wilcoxon test,
- Restricted mean survival time (RMST)^33^) using the χ^2^ test. If significant differences had been detected in the RMST, homogeneous groups were determined using the χ^2^ test for each pair of experimental combinations using the Bonferroni correction for multiple tests. Finally, survival curve estimators and limited median survival time were also determined.

### Ethical approval

Experimental research on animals (fruit flies) complied with the relevant institutional, national, and international guidelines and legislation.

## Results

The results show the effects of experimental treatments on two indicators: the lifespan and the body mass of the model organisms.

### Average lifespan and survival curves

For both sexes of *Drosophila melanogaster*, the average lifespan differed significantly between the experimental combinations (Table 2). Different responses of males and females to the use of chips (inductors) were also observed. More precisely, the following conclusions can be drawn.

**Table 2:** Analysis of the average fruit fly lifespan (days) in 4 experimental treatments.

Treatments	Females		Males	
	Mean	Standard deviation	Mean	Standard deviation
SiO_2_	47.36 a	10.23	42.27 ab	12.61 a
Si^0^	35.31 ab	11.07	32.73 b	14.63 a
Blind test	33.13 b	11.81	41.53 ab	20.20 a
Control test	39.38 ab	11.28	48.50 a	7.56 a
Results of analysis of variance	F = 4.37; p = 0.0086		F = 4.92; p = 0.0054	

The value of the test statistic F and the p-value test of the variance analysis are given in the last row of the table. Differences between treatments were compared using Tukey's HSD test. Different lowercase letters in columns (a and b) indicate statistically different values. The blocking effect was insignificantly different.

Females: The life expectancy was extended by single-crystal silicon dioxide (SiO_2_) compared to single-crystal silicon (Si^0^) and blank. In the control sample, an intermediate lifespan was observed between the highest SiO_2_ and the lowest of Si^0^ as the well as blank.

Males: The average lifespan of males was reduced under the influence of Si^0^ compared to the blind tested lifespan of fly. In the case of the control and SiO_2_, the differences in results were insignificant.

**Figure 1:**
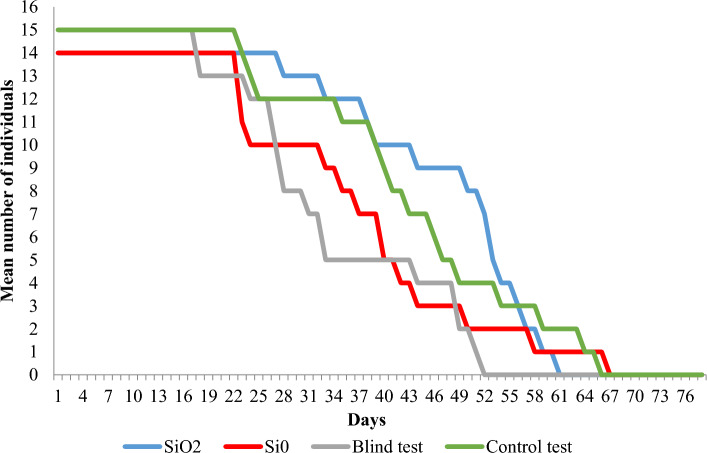
Estimates of female fruit fly survival curves over time (days) in 4 experimental treatments.

**Figure 2:**
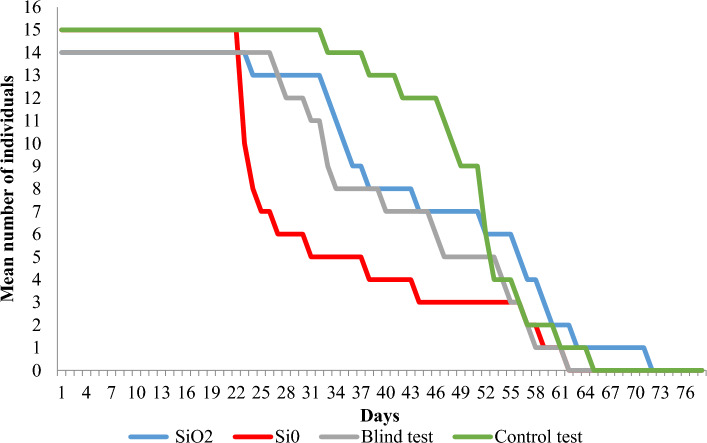
Estimates of male fruit fly survival curves over time (days) in 4 experimental treatments.

Estimated survival curves are shown in Figs. 1 and 2. These curves appear to be remarkably different for various experimental treatments. The Wilcoxon test shows significant differences for both males and females (Table 3). The log-rank test confirms the significance of differences only in the case of females. On the contrary, for males, the p-value is slightly higher than the standard significance level of 0.05. However, by increasing this level to 0.1, the null hypothesis of the equality of the three survival curves is already rejected. In conclusion, it was found that the survival of males and females in terms of survival curves differed significantly between treatments.

**Table 3:** Results of logarithmic rank (log-rank) and Wilcoxon tests of equality of fruit fly survival curves (column χ^2^- values of test statistics; column DF—degrees of freedom).

	Females			Males		
	χ^2^	DF	*p*	χ^2^	DF	*p*
log-rank	10.7182	3	0.0134	7.1406	3	0.0675
Wilcoxon	11.5729	3	0.0090	15.2483	3	0.0016

Females: A single crystal of silicon dioxide (SiO_2_) has been shown to have a positive effect on fly survival. The evaluation of the equality of survival curves showed differences between combinations at the significance level of *p* = 0.0006 (Fig. 1, Table 3). Survival in SiO_2_ and control treatments was significantly higher compared to Si^0^ and blank (Fig. 1, Table S1).

Males: The single crystal of Si^0^ significantly reduces the survival rate of male fruit flies compared to the control sample. For quartz (SiO_2_) and blank (Alº) survival was intermediate (Table 3, Fig. 2, Table S1).

### Mean body mass

For both sexes, a similar influence of chip inductors on body weight was observed (Table 4):

**Table 4:** Average body mass of female and male fruit flies (g) in 4 experimental treatments.

Treatment	Females		Males	
	Mean	Standard deviation	Mean	Standard deviation
(SiO_2_)	0.00100 a	0.00048	0.00130 a	0.00190
(Si^0^)	0.00083 a	0.00039	0.00074 b	0.00085
Blind test	0.00077 a	0.00048	0.00060 ab	0.00029
Control test	0.00094 a	0.00059	0.00055 ab	0.00023
Results of the analysis of variance	F = 2.80; p = 0.053		F = 3.20, p = 0.035	

Body masses were compared using the analysis of variance at the significance level of p = 0.05. Differences between treatments were compared using Tukey's HSD test. Different lowercase letters in columns (a and b) indicate statistically different values. The blocking effect was not significantly different.

Males: There were significant differences in the body mass of the males between the experimental treatments (ANOVA, p = 0.035) (Table 4). The highest body mass was measured in treatment with a single crystal of silicon dioxide (SiO_2_) compared to a single crystal of silicon (Si^0^). Other combinations (blind and control) were insignificantly different.

Females: A similar trend was demonstrated (statistically insignificant results)—increased body mass under the influence of SiO_2_ (and additionally the control) compared to the influence of Si^0^ (Table 4).

## Discussion

The Boltzmann–Gibbs entropy as a measure of the degree of system disorder and energy dissipation is related to the concept of metabolic stability and homeostasis of organisms^3^. Moreover, the Gibbs entropy of a system with phase space *χ*, for example *χ* = ${\mathbb{R}}$^*6N*^ = {(*q*_*1*_,*v*_*1*_, … ,*q*_*N*_,*v*_*N*_)} for *N-point* particles in ${\mathbb{R}}$^3^ with positions *q*_*j*_ and velocities *vj* is as follows:

1 $$ S_{G} \left( \rho \right) = - k\int\limits_{\chi } {dx \rho \left( x \right) \ln \left[ {\rho \left( x \right)} \right]} $$

where *k* is the Boltzmann constant, *dx* = *N!*^−1^*d*^3^*q*_*1*_* d*^3^*v*_*1*_*… d*^3^*q*_*N*_* d*^3^*v*_*N*_ is the phase space volume measure, *ln* is the natural logarithm, and *ρ*—a probability density on *χ*.

In turn, the Boltzmann entropy of a system is defined as follows:

2 $$ S_{B} \left( X \right) = k\ln \left[ {vol \Gamma \left( X \right)} \right] $$

where X ∈ *χ* is the actual phase point of the system, vol means the volume in, and Γ(X) is the set of all phase points^34^. The equivalence of these two entropies is mathematical, but not physical. The Boltzmann entropy (S_*B*_) is proper for isolated (microcanonical) systems, while the Gibbs entropy (S_*G*_) is canonical and exchanges energy with the surrounding. In other words, the Gibbs entropy is the generalisation of the Boltzmann entropy for all systems, whereas the Boltzmann entropy describes only the entropy of the system remaining in global thermodynamic equilibrium. It is known that entropy is essential in the prediction of the extent and direction of complex chemical reactions. The Gibbs free energy change of the system: ΔG = ΔH–TΔS, where ΔH is the enthalpy change and ΔS is the entropy change at given temperature T (in Kelvin scale).

The enzymatic reactions of ectotherms differ from those of endotherms in the magnitudes of the following thermodynamic parameters: free energy of activation, enthalpy of activation, and entropy of activation. Furthermore, the relative importance of the enthalpic and entropic contributions to the free energy of activation is different for these two classes of organisms ^8^. Furthermore, metabolic stability or the ability of cells (organisms) to maintain fixed values of redox pairs in the face of random fluctuations in the rate of enzymatic reactions could be determined as the longevity of a cell (organism). The stability-longevity hypothesis assumes that age-related loss of function results from the dysregulation of the steady-state values of redox pairs and coexisting disturbances in homeostasis. Thus, with ageing, the entropy of the organism increases, i.e. the level of disorder^35^. In the case of ectotherms, no study has yet been reported on the effect of entropy change on their metabolism, confirming the innovative nature of our research. Ambient temperature is known to influence the lifespan of ectothermic animals, including the fruit fly *Drosophila melanogaster*^22,36^. However, temperature is only one of the state parameters, along with entropy^37^. The effect of temperature on the survival and metabolic rate of adult fruit flies was studied in detail^22^. Based on the results of these studies, the average lifespan of adults was approximately 50% shorter at 298.15 K and 1 bar than that achieved at 293.15 K. An analogous difference in lifespan has been observed for other temperatures of 298.15 and 301.15 K. At the highest temperature considered (301.15 K), the average lifespan was only 25 days, i.e., 4 times smaller than at 293.15 K (100 days). In other studies, the increase in temperature also caused an acceleration of metabolism, which was correlated with a shorter lifespan^22,38^. The lifespan values presented in^22^ are comparable to the results of this research. The average lifespan of the flies reared at 296.15 K was 39 days for females and 48 days for males, thus corresponding to the lifespan values reported in^22^ at temperatures between 293.15 and 298.15 K.

In this research, it was assumed that the reduction in entropy extends the lifespan of the fruit fly indirectly because of the slowing of its metabolism indirectly. The experiment was carried out isothermally (296.15 K) and therefore the influence of the temperature itself on the tested organisms was eliminated. In this research, it was shown that reduction in entropy by the influence of a single crystal of silicon dioxide (SiO_2_) resulted in an extension of the lifespan of females, while a single crystal of silicon (Si^0^) caused a shortening of the lifespan of males. The reactions of males and females were different, which may be due to many aspects, for example, differences in body fat content depending on sex^39^. In the case of females, it can be assumed that the reduction in entropy with the use of the SiO_2_ resulted in a decrease in the metabolic rate, comparable to the effect observed at 293.15 K in the study reported in^22^ and, consequently, in life extension. In the case of exposure to Si^0^, increased mortality in the population of fruit flies. Si^0^ resulted in an almost twice higher reduction of water entropy (ΔS = 51 J K^−1^ mol^−1^) compared to the SiO_2_ (ΔS = 28 J K^−1^ mol^−1^). Such an effect of lowering the entropy too much may be like reaching a state at the limit (or below) of the so-called physiological zero for this species, which is around 278.15 K^40^.

In terms of changes in the mass of organisms subjected to entropy manipulation, it has been shown that by lowering the entropy of the aquatic environment, thus supporting the processes of homeostasis energetically by reducing the production of entropy, additional effects can be obtained in the form of an increase in the body mass of birds or breeding mammals, but only in the period after puberty, i.e. in the period when genetic factors are no longer decisive for the speed of changes in energy metabolism^41^. In the case of ectotherms, size/mass is directly related to the metabolic rate^42^. The rule of dependence of body size on ambient temperature (TSR) explains that at higher temperatures ectotherms achieve smaller body sizes, which is associated with an increase in metabolic rate^42^. Therefore, in the case of the studies performed, the reduction in entropy should decrease the metabolic rate and increase the body mass of the flies.

At the same time, in connection with this, an increase in body mass under the influence of entropy reduction should be a positive phenomenon. These assumptions were confirmed in the case of male flies, whose body mass achieved was significantly higher in combination with SiO_2_ than for the Si^0^, blank, and control. The results of this research are consistent with those obtained in work^22^, in which an increase in temperature caused an increase in metabolism and a decrease in the body mass. With respect to the adopted hypotheses, the life span and body mass of the test organisms can be considered as a reaction to a change in the rate of metabolism.

## Conclusions

This work presents the results of the first experimental study that confirms the effect of changes in entropy (under constant other external conditions) on the metabolism of a living organism. Studies showed that, on the one hand, the reduction of the Boltzmann–Gibbs entropy under isothermal conditions (296.15 K and 1 bar) in the aquatic environment by the optimal value of ΔS = 28.49 J K^−1^ mol^−1^ results in an extension of the lifespan of female fruit flies. On the other hand, an excessive decrease in the Boltzmann–Gibbs entropy of the aquatic environment by ΔS = 51.14 J K^−1^ mol^−1^ results in a shorter life of the fruit fly males, probably due to an excessive reduction in their metabolism. The body mass of the flies at the time of death of the organism turned out to be a specific reaction of metabolism to changes in the entropy of the aquatic environment. Furthermore, the increase observed in the body mass of female and male fruit flies due to the decrease in entropy by the optimal value of ΔS = 28.49 J K^−1^ mol^−1^ is a positive phenomenon. In summary, the isothermal changes of the entropy itself (along with the temperature) can affect the metabolism and, thus, also the lifespan of ectothermic organisms and their mass gain. This phenomenon requires further research in this area, both on ectotherms and endotherms. The findings are promising for further applications to extend the life of other more complex living organisms, including human life.

### Supplementary Information

Supplementary Information.

## Data Availability

The data sets generated during and/or analysed during the current study are available from the corresponding author on reasonable request.
