# Supplementary material for: The isothermal Boltzmann–Gibbs entropy reduction affects survival of the fruit fly Drosophila melanogaster
Source: Sci Rep. 2023 Aug 29;13:14166. doi: 10.1038/s41598-023-41482-x (PMC10465501; doi:10.1038/s41598-023-41482-x)
Supplement: Supplementary file 1 — Supplementary Information. [file 41598_2023_41482_MOESM1_ESM.docx]

**Figure S1**.

a) The experimental design of a randomized block system in 5 blocks. The minimum distance between each of the neighboring groups was 40 cm.

b) The exemplary flasks arrangement within each group: 3 flasks with males (triangles) and 3 flasks with females (circles) were drawn.

a)

b)


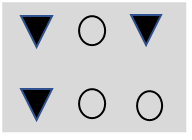


**Table S1**. χ^2^ test results of the fruit fly limited survival curves for each pair of experimental treatments for females and males.

|  | | Females | | | Males | | |
| --- | --- | --- | --- | --- | --- | --- | --- |
| Treatments comparison | | Diff . | χ^2^ | p | Diff . | χ^2^ | p |
| SiO_2_ | Si^0^ | 11.60 | 12.46 | 0.0004 | 12.83810 | 6.6936 | 0.0097 |
| SiO_2_ | Blind test | 9.131 | 8.62 | 0.0033 | 4.038095 | 0.7929 | 0.3732 |
| SiO_2_ | Control test | 0.66 | 0.051 | 0.8207 | -3.76190 | 0.9276 | 0.3355 |
| Si^0^ | Blind test | -2.47 | 0.541 | 0.4620 | -8.80000 | 3.4295 | 0.0640 |
| Si^0^ | Control test | -10.94 | 11.72 | 0.0006 | -16.6000 | 15.9539 | <.0001 |
| Blind test | Control test | -8.47 | 7.90 | 0.0050 | -7.80000 | 4.6043 | 0.0319 |
